# Construction of a risk model and prediction of prognosis and immunotherapy based on cuproptosis-related LncRNAs in the urinary system pan-cancer

**DOI:** 10.1186/s40001-023-01173-9

**Published:** 2023-06-27

**Authors:** Zhihui Ma, Haining Liang, Rongjun Cui, Jinli Ji, Hongfeng Liu, Xiaoxue Liu, Ping Shen, Huan Wang, Xingyun Wang, Zheyao Song, Ying Jiang

**Affiliations:** Mudanjiang Medical University, Mudanjiang, Heilongjiang China

**Keywords:** Urinary system pan-cancer, Cuproptosis, LncRNA immunotherapy, Cell death, Tumor

## Abstract

**Background:**

Urinary pan-cancer system is a general term for tumors of the urinary system including renal cell carcinoma (RCC), prostate cancer (PRAD), and bladder cancer (BLCA). Their location, physiological functions, and metabolism are closely related, making the occurrence and outcome of these tumors highly similar. Cuproptosis is a new type of cell death that is different from apoptosis and plays an essential role in tumors. Therefore, it is necessary to study the molecular mechanism of cuproptosis-related lncRNAs to urinary system pan-cancer for the prognosis, clinical diagnosis, and treatment of urinary tumors.

**Method:**

In our study, we identified 35 co-expression cuproptosis-related lncRNAs (CRLs) from the urinary pan-cancer system. 28 CRLs were identified as prognostic-related CRLs by univariate Cox regression analysis. Then 12 CRLs were obtained using lasso regression and multivariate cox analysis to construct a prognostic model. We divided patients into high- and low-risk groups based on the median risk scores. Next, Kaplan–Meier analysis, principal component analysis (PCA), functional rich annotations, and nomogram were used to compare the differences between the high- and low-risk groups. Finally, the prediction of tumor immune dysfunction and rejection, gene mutation, and drug sensitivity were discussed.

**Conclusion:**

Finally, the candidate molecules of the urinary system pan-cancer were identified. This CRLs risk model may be promising for clinical prediction of prognosis and immunotherapy response in urinary system pan-cancer patients.

## Introduction

The pan-cancer study aims to identify similarities and differences between tumors from the perspective of genome, transcriptome, proteome, epigenome, and other multi-structured data, thus guiding clinical diagnosis, prognosis, and treatment options. Pan-cancer of the urinary system is a general term for malignancies of the urinary system, including renal cell carcinoma (RCC), prostate cancer (PRAD), and bladder cancer (BLCA) [1]. Urological malignancy is commonly worldwide, characterized by difficulties in early diagnosis, multiple postoperative metastases, tumor heterogeneity, and insensitivity to chemotherapy drugs [2, 3]. According to the global cancer statistic in 2020, there were more than 2.4 million new cases of urinary tract tumors, accounting for 12.5% of cancer incidence and 7.7% of new cancer deaths worldwide [4].

Cuproptosis is a newly defined form of cell death that binds lipoic acid to substrate proteins in the mitochondria through lipoylation, causing lipoacylated mitochondrial enzymes to accumulate in the mitochondria in a toxic manner. These enzymes simultaneously inhibit multiple lipoacylases and copper-binding enzymes from gaining new cellular activity, resulting in cell death [5]. This type of death is closely associated with carcinogenesis, neurological disease, and genetic disorders, such as Menkes and Wilson’s diseases [6–8]. Cuproptosis has unique morphological and bioenergy characteristics that can be easily distinguished from other types of programmed cell death, such as apoptosis or ferroptosis [9]. Currently, cuproptosis is considered a promising therapeutic strategy for cancer, especially for cancers with rapid respiratory rate [9]. In the urinary system pan-cancer, although cuproptosis is extremely promising, the pattern of copper metabolism in tumor treatment is still not entirely clear. According to recent studies, immunotherapy of tumors is another effective treatment for tumors following surgery, radiotherapy, chemotherapy, and targeted treatment. For example, the application of immune checkpoint PD1/PD-L1 inhibitors in clinical treatment increases antitumor immunity in patients. Hence, we performed this study for a comprehensive analysis of cuproptosis and immunotherapy in the urinary system pan-cancer.

LncRNA is a class of long non-coding RNA whose transcript length exceeds 200nt which does not participate in gene coding [10]. However, lncRNAs are reported to modulate tumor growth, progression, and metastasis, and have been implicated as potential alternative biomarkers and therapeutic targets for cancer [11–13]. For instance, LncRNA ANRIL is upregulated in hepatocellular carcinoma (HCC) and promotes the proliferation and mitochondrial function of HCC by regulating Mir-199a-5p/ARL2 axis [14].

However, the urinary system has identified only a few cuproptosis-related therapeutic targets. So, further clinical sample-based screenings for cuproptosis-related genes (CRGs) are necessary for urinary system diagnoses and treatments [15]. In our study, 12 cuproptosis-related lncRNAs from urinary system pan-cancer were identified and a prognostic prediction model was constructed using the machine learning method. The scientific of the model was evaluated through survival analysis, principal component analysis, tumor immunity, and drug sensitivity analysis, which provides a new method for clinical diagnosis and treatment of urinary system tumors in the future.

## Materials and methods

### Data downloaded and processed

We downloaded the transcriptome profiling data of three urinary system carcinomas from The Cancer Genome Atlas (TCGA) database (https://portal.gdc.cancer.gov/), including 128 normal and 893 tumor samples in RCC, 499 normal and 52 tumor samples in PRAD, 414 normal and 19 tumors samples in BLCA. Using Perl and R language extracted the LncRNA from each of these three kinds of tumors. In addition, we downloaded the clinical data of patients with three urinary tumors from the TCGA database.

### Screening of co-expression CRLs

According to previous studies, 19 cuproptosis-related genes were collected from PubMed (https://pubmed.ncbi.nlm.nih.gov), which have been reported to be associated with cuproptosis. Then, Pearson’s correlation analysis was used to calculate the correlation between CRGs and lncRNAs in three urinary system tumors. The square of correlation coefficient |R^2^|> 0.4 and *P* < 0.001 was considered to be CRLs. Then, co-expressed CRLs were got by the intersection of lncRNAs of those using an upset diagram. Finally, we combined and normalized the expression values of co-expressed CRLs samples from the three urinary system tumors for the future comprehensive analysis of urinary system pan-cancer.

### Establishing and validating a prognostic model

The entire TCGA cohort was randomized as a training cohort and a testing cohort at radio 1:1. First, univariate Cox regression analysis was used to screen LncRNAs with overall survival, of which *P* value < 0.05 were identified as survival-related CRLs. Next, survival-related CRLs were introduced into the least absolute shrinkage and selection operator (Lasso) regression and multivariate Cox regression analysis to finally established a prognostic model. We used the constructed risk model and the calculation formula to calculate the risk score of all patients and the calculation formula was as follows: Risk score = ∑(Expi*Coefi).

Subsequently, all patients were stratified into high-risk and low-risk groups using the median risk score. Kaplan–Meier survival curves for overall survival (OS) and progression-free survival (PFS) between the high-risk and low-risk groups in the training cohort were compared using the “survival” package. The receiver operating characteristic (ROC) curve analysis was used to predict the performance of prognostic factors in terms of the OS training cohort. To validate the risk model, the KM survival analysis and ROC curve analysis were also applied in the testing cohort and the entering cohort. To further validate the risk model, the “Scatterplot3D” package was used for efficient dimensionality reduction, grouping, and visualization based on data from all gene expression profiles, CRGs, CRLs, and risk model.

### Exploration of the clinical and predictive value of the prognostic risk model

Age, gender, T stage, and risk score were included in the univariate and multivariate Cox regression analysis to identify independent risk factors associated with the prognosis. To better predict the prognosis, we used the “regplot” package to construct the nomogram which can be used to predict the survival of 1-, 3-, and 5 years. The analysis of time-dependent consistency index (C-index) and ROC curve were used to check the accuracy of the model. Finally, calibration plot was generated using the “rms” package which showed the consistency of the predicted events at 1, 3 and 5 years with the true outcomes.

### Functional analysis

The limma package was used to get the differentially expressed genes between the high- and low-risk groups. The DEGs were identified as logFC > 1 and *P* value < 0.05. Kyoto Encyclopedia of Genes and Genomes (KEGG) pathway and Gene Ontology (GO) analyses were performed for genes enriched in the low- and high-risk categories using the R package “clusterProfiler”.

### Exploration of the risk model in the immunotherapeutic treatment and drug-sensitive analysis

The number of mutations and neoantigens and immunophenoscore (IPS) information was got from TICA (https://tcia.at/home). Tumor immune dysfunction and exclusion (TIDE) scores, immune exclusion score, and T cell dysfunction score were from the TIDE portal (http://tide.dfci.harvard.edu/login/) based on the normalized transcriptome data of three urinary system carcinomas from TCGA. We downloaded the mutation data of three urinary system carcinomas from the TCGA database. The map tools package was used to calculate the tumor mutation burden (TMB). Immune score, ESTIMATE score, and stromal score were acquired using the ESTIMATE algorithm [16]. Generally speaking, the stromal score is positively correlated with the immune score. The ESTIMATE score can be used to evaluate tumor purity. The higher the ESTIMATE score, the lower the tumor purity. Next, estimating the Proportions of Immune and Cancer cells (EPIC) algorithm was used to calculate the proportion and distribution of different types of immune cells in different risk groups [17]. The GSVA analysis was performed through the “GSVA” package to explore the immune function of high- and low-risk groups. We used the MCP algorithm and GSEA to confirm the immune cell infiltration. The “pRRophetic” package was used to calculate the half-maximal inhibitory concentration (IC50) and compare the sensitivity between high-risk and low-risk groups [18].

### Statistical analysis

All statistical analyses used the R language (version 4.1.3) and Perl language (https://www.perl.org).

## Results

### Identification of cuproptosis-related lncRNAs in urinary system pan-cancer

**Fig. 1:**
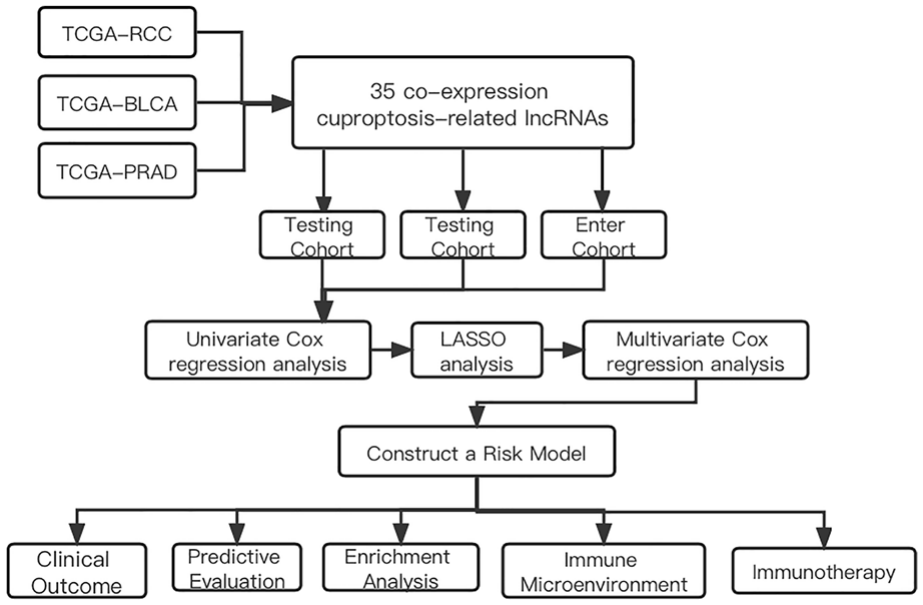
Flowchart of the whole study

**Fig. 2:**
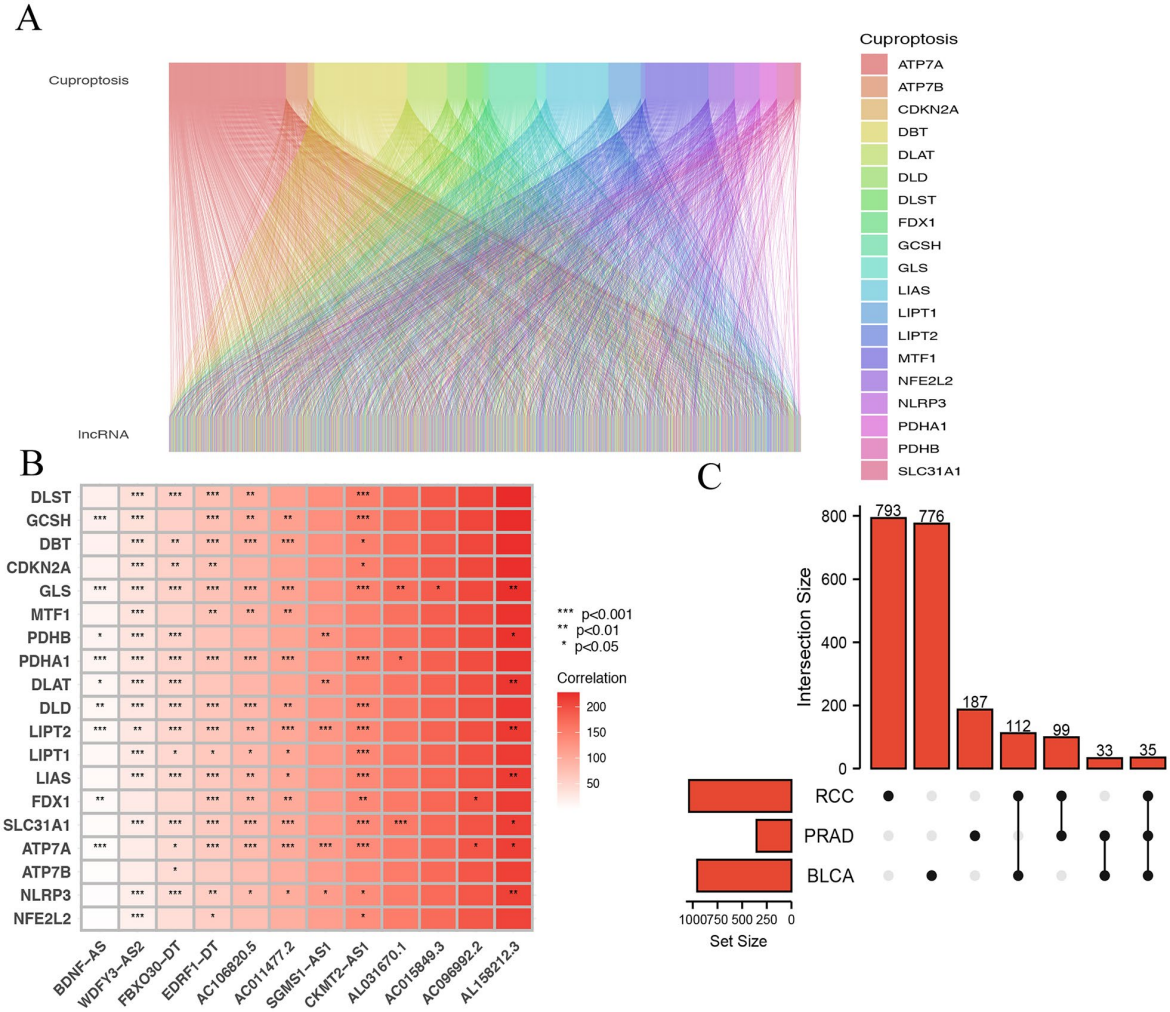
Identification of co-expression CRLs in urinary system pan-cancer. **A** The relationship between 18 CRGs and LncRNAs. **B** The correlation between 18 CRGs and the 11 CRLs. **C** 35 co-expressed CRLs in the urinary system

The detailed workflow for prognostic risk model construction and subsequent analyses is shown in Fig. 1. We collected 19 CRGs from PubMed which had been reported. 776 lncRNA was identified in BLCA, 178 lncRNA was identified in PRAD and 793 lncRNA was identified in RCC for Pearson correction analysis. All CRLs co-expression network was visualized using the Sankey diagram (Fig. 2A). The correlation between CRGs and some CRLs in the three urinary system tumors is shown in Fig. 2B. At last, there were 35 co-expressed CRLs in the three urinary system tumors (Fig. 2C).

### Construction and validation of the prognosis risk model

**Fig. 3:**
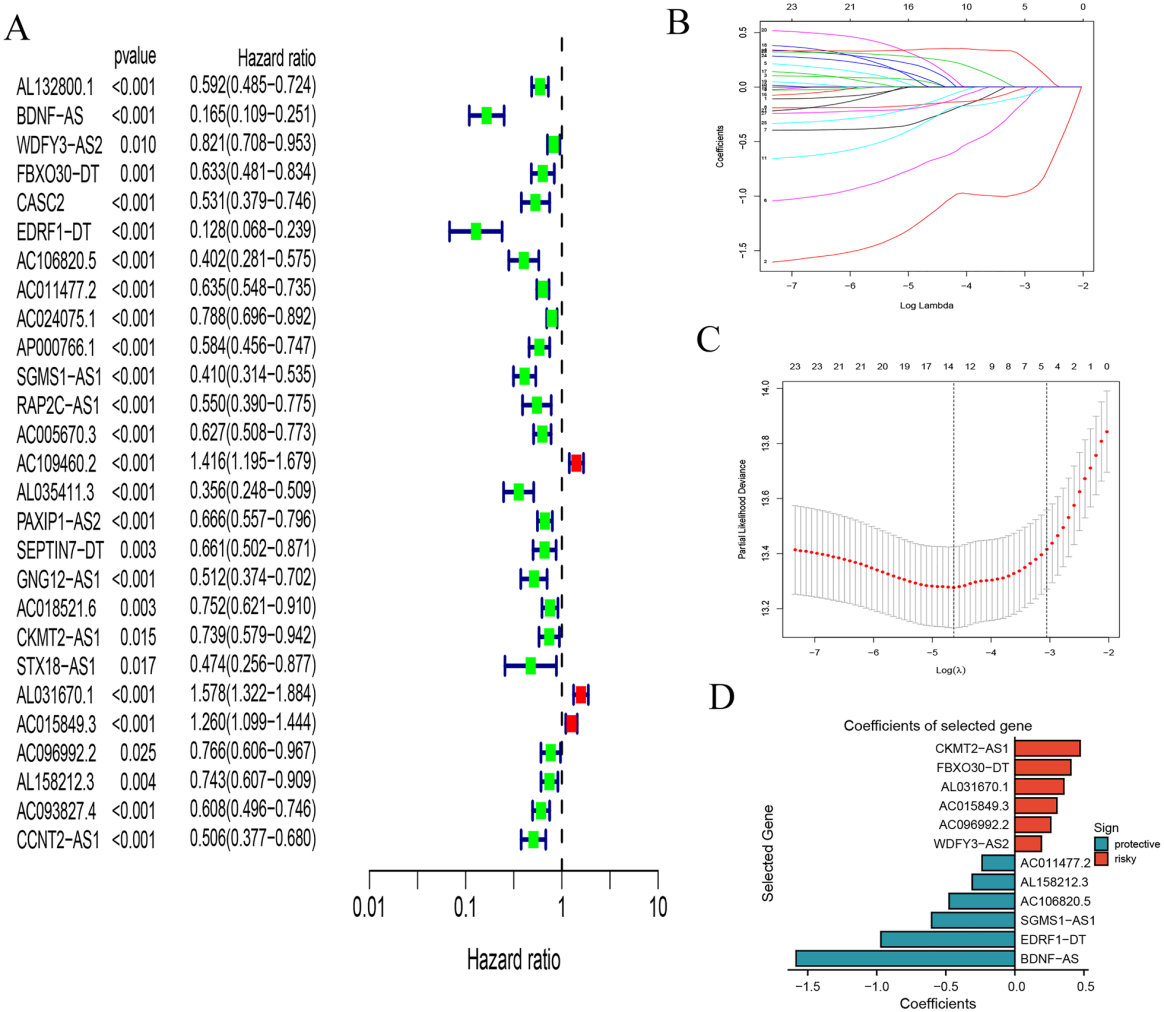
Construction and validation of a risk model. **A** Univariate Cox analysis revealed 28 prognostic lncRNAs associated with OS. **B**, **C** Lasso analysis. **D** Coefficients of selected genes

Univariate Cox regression analysis showed that 28 CRLs were significantly correlated with overall survival in urinary system pan-cancer (*P* < 0.05, Fig. 3A). 28 CRLs associated with overall survival were subjected to lasso analysis to obtain the best risk markers for assessing the prognosis of patients with urologic pan-cancer. We performed multivariate Cox regression and screened 12 lncRNAs as independent factors including BDNF-AS, WDFY3-AS2, FBXO30-DT, EDRF1-DT, AC106820.5, AC011477.2, SGMS1-AS1, CKMT2-AS1, AL031670.1, AC015849.3, AC096992.2, AL158212.3 (Fig. 3D). At the same time, we got coefficients of each lncRNA with multivariate cox regression. We could calculate each patient’s risk scores using the selected gene expression data and coefficients. The calculation formula was as follows:

Risk score = coef_BDNF-AS_ * exp_BDNF-AS_ + coef _WDFY3-AS2_* exp_WDFY3-AS2_ + coef_FBXO30-DT_*exp_FBXO30-DT_ + coef_EDRF1-DT_*exp_EDRF1-DT_ + coef_AC106820.5_*exp_AC106820.5_ + coef_AC011477.2_*exp_AC011477.2_ + coef_SGMS1-AS1_*exp_SGMS1-AS1_ + coef_CKMT2-AS1_*exp_CKMT2-AS1_ coef_AL031670.1_*exp_AL031670.1_ + coef_AC015849.3_*exp_AC015849.3_ + coef_AC096992.2_*exp_AC096992.2_ + coef_AL158212.3_*exp_AL158212.3_.

**Fig. 4:**
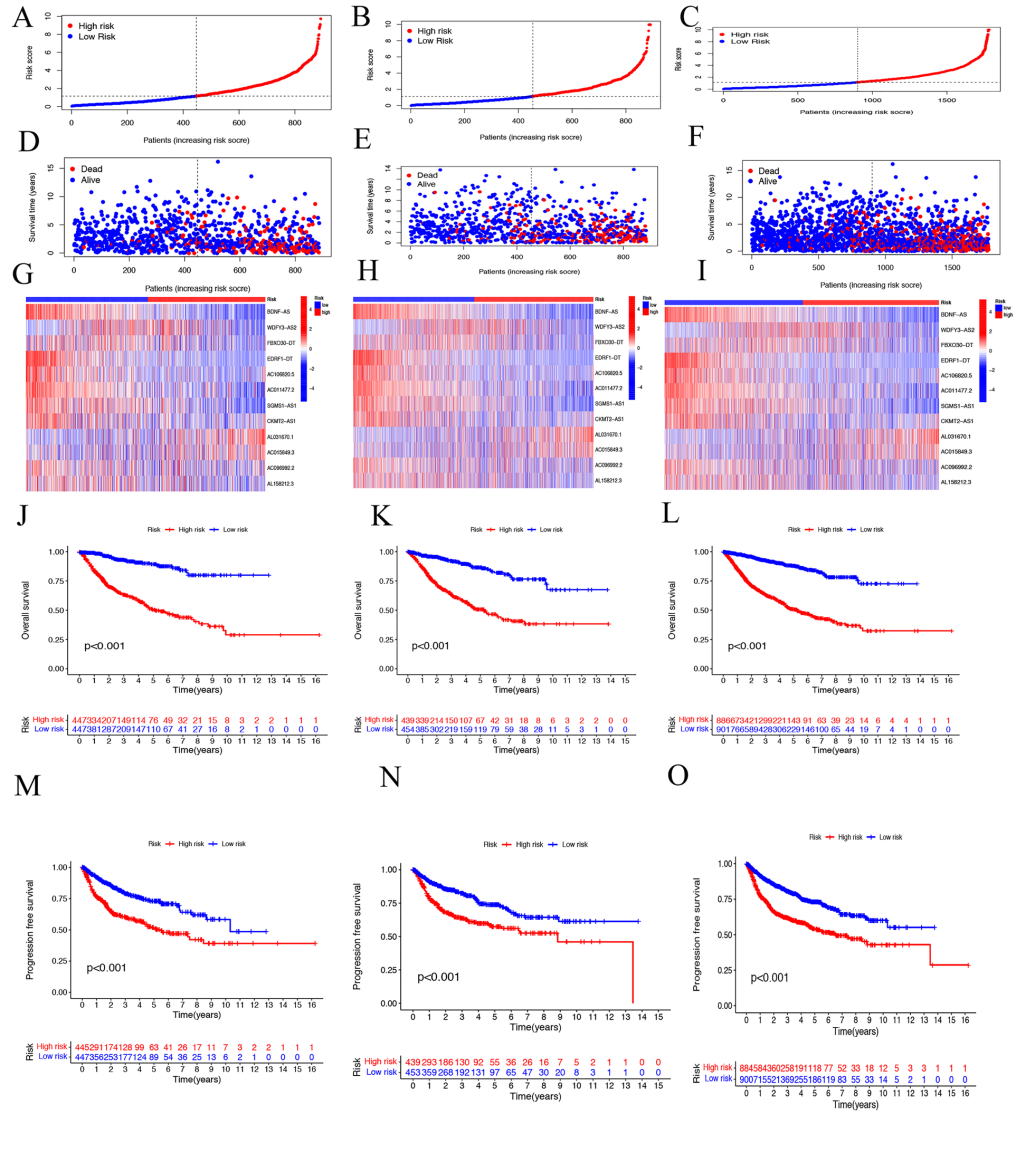
The assessment of the risk model for prognostic value. Distribution of patient risk classes in the training cohort (**A**), testing cohort (**B**), and enter cohort (**C**). Survival state of patients in the training cohort (**D**), testing cohort (**E**), and enter cohort (**F**). The expression of prognosis CRLs in the training cohort (**G**), testing cohort (**H**), and enter cohort (**I**). **J**–**L** Kaplan–Meier curves of the OS of patients in the high- and low-risk groups in the training cohort, testing cohort and enter cohort. M–O Kaplan–Meier survival curves of the PFS of patients in the high- and low-risk groups in the training cohort, testing cohort, and enter cohort

Next, we used the median to categorize all patients into high-risk and low-risk groups. Patients with different risk grades in the training, testing, and entering cohorts are shown in Fig. 4A–C. Survival state in three groups is shown in Fig. 4D–F. The death rate of the high-risk group was higher than that of the low-risk group. Heatmap was shown the expression of selected genes in high- and low-risk groups (Fig. 4G–I).

To further validate the risk model, we performed the OS and PFS survival analysis of urinary pan-cancer. The KM curves showed that the OS and PFS of patients in the low-risk group were significantly higher than those in the high-risk group in the training cohort, testing cohort, and enter cohort (Fig. 4J–O).

### Principal component analysis (PCA) between high- and low-risk groups

**Fig. 5:**
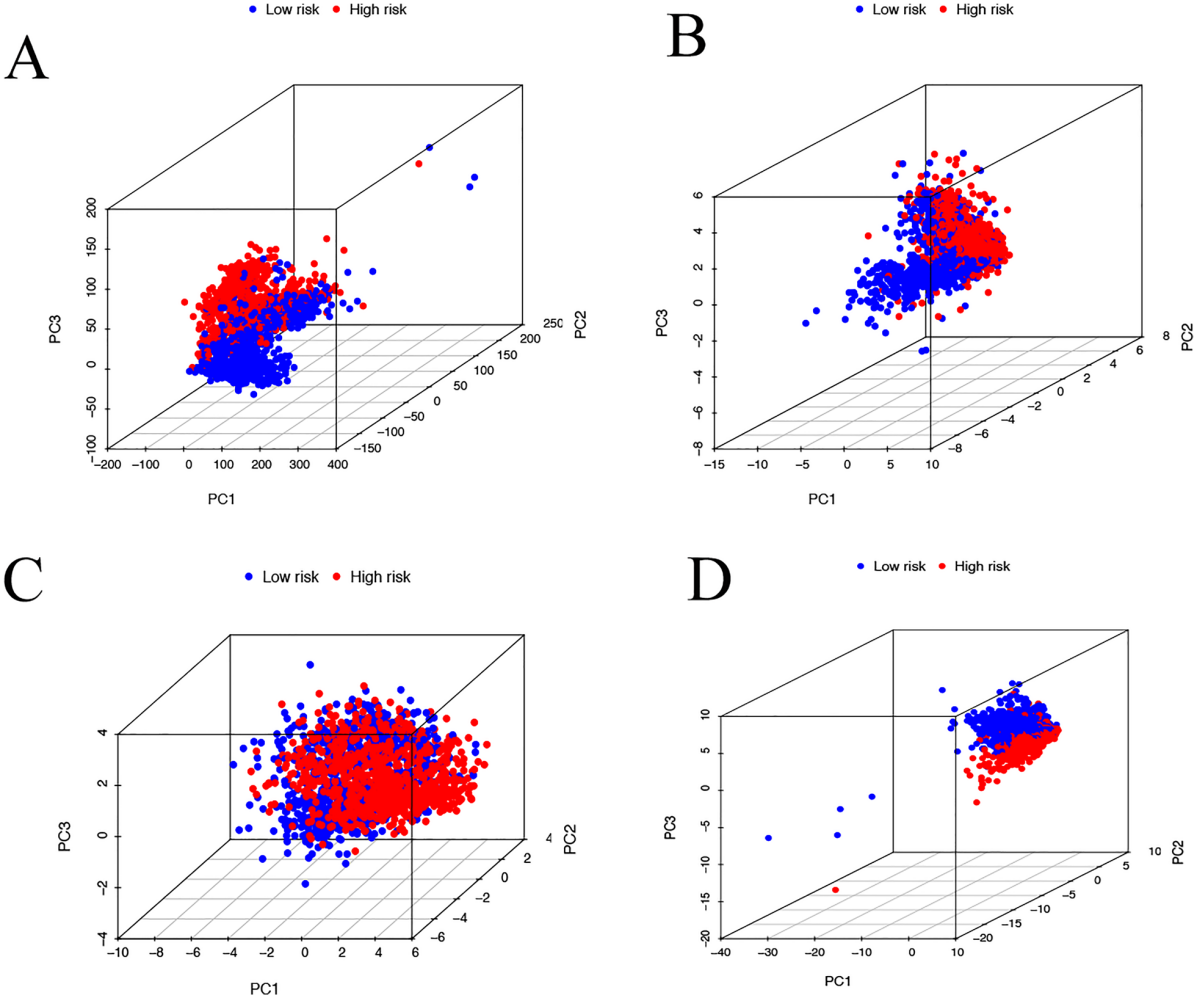
Principal component analysis between the high- and low-risk groups. **A** entire gene expression profiles, **B** cuproptosis-related genes, **C** CRLs, and **D** risk model based on the representation profiles of the 12 cuproptosis-related lncRNAs in the TCGA entire set

To verify the grouping ability of the CRLs risk model, PCA analysis was performed to detect the difference between high- and low-risk groups based on the standardized gene expression data for the urinary system pan-cancer. Figure 5A–C shows the PCA results based on all gene expression profiles, CRGs, CRLs, and risk model classified by the expression profiles of the 12 CRLs. PCA analysis showed that patients in different risk groups were distributed in two directions (Fig. 5D). These results suggest that our model can distinguish between low and high-risk groups.

### Prognostic value of the clinical feature based on the prognosis risk model and construction of nomogram

**Fig. 6:**
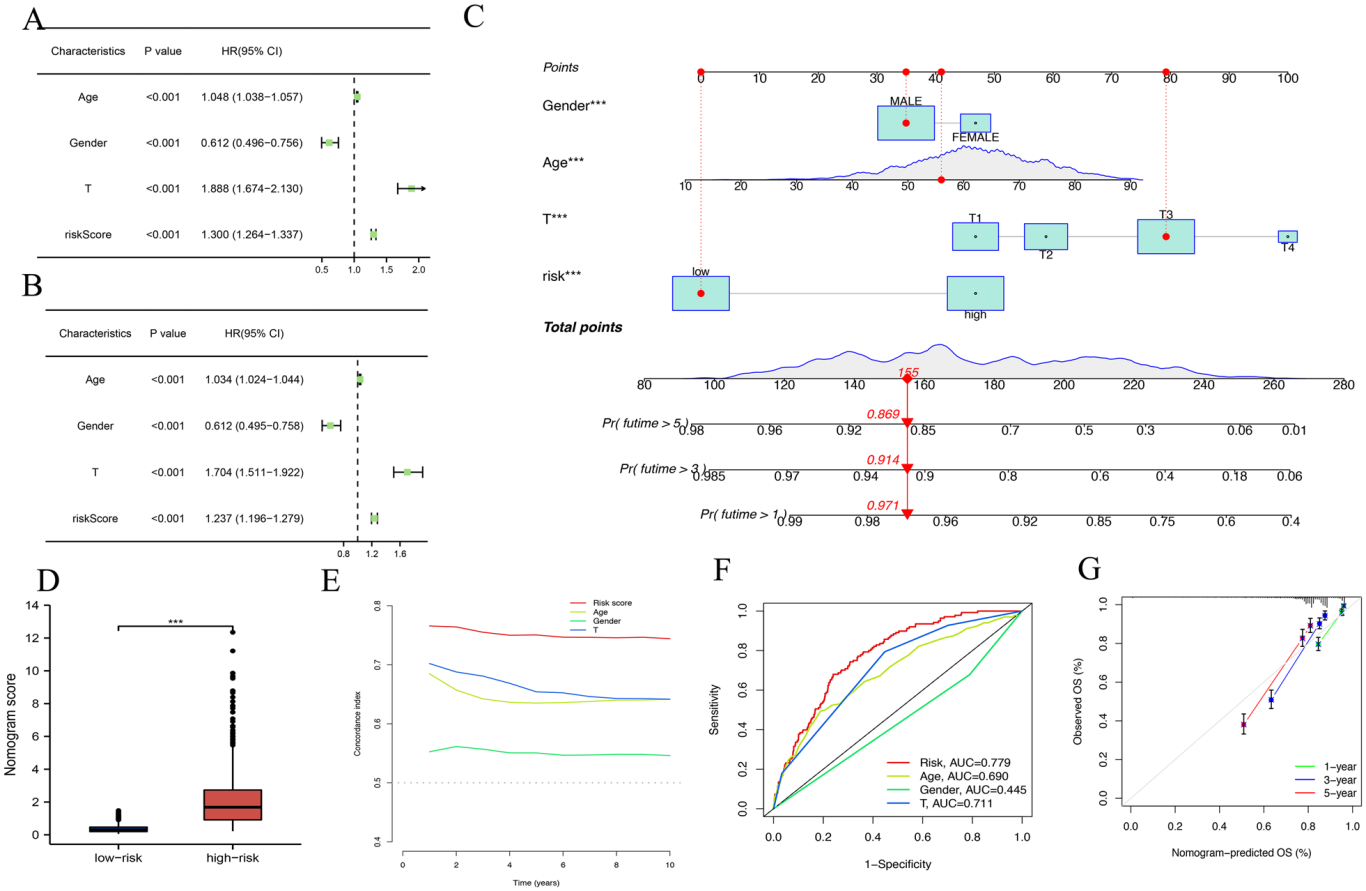
Construction and evaluation of a prognostic nomogram. **A** Univariate Cox regression analysis of clinical characteristics. **B** Multivariate Cox regression analysis of clinical characteristics. **C** The nomogram predicts the probability of the 1-, 3-, and 5-year OS. **D** boxplot of nomogram score between high- and low-risk groups. **E** Consistency index of clinical characteristics. **F** ROC curves of clinical characteristics. **G** Calibration curves of the 1-, 3-, and 5-year OS

We extracted the patients' age, gender, and T stage from clinical information and introduced them in the univariate and multivariate Cox regression analysis. We determined age, gender, T stage, and risk scores as independent prognostic factors (Fig. 6B). Next, nomograms containing risk ratings and independent prognostic factors were produced to predict 1-, 3-, and 5-year OS incidence (Fig. 6C). We have created a clearer online website to make our predictive model easier to use in the clinic (https://l5035t-zhihui-ma.shinyapps.io/Urinarysystem/). The red line indicated information from the 20th patient and Nomo's score and 1, 3, and 5-year OS incidence. Nomo's score in the low-risk group was lower than in the high-risk group (Fig. 6D). The AUC of risk score was the biggest of all factors. Consistency index and ROC analysis were conducted to predict the uniqueness and susceptibility of risk scores in predicting the prognosis of urinary system patients. The conformance index of the risk score and the area under the ROC curve (AUC) was the highest in risk scores (Fig. 6E, F). Calibration curves displayed that the observed versus predicted rates of the 1-, 3-, and 5-year OS revealed ideal consistency (Fig. 6G).

### Exploration of the correction of immune landscape with prognostic risk model

**Fig. 7:**
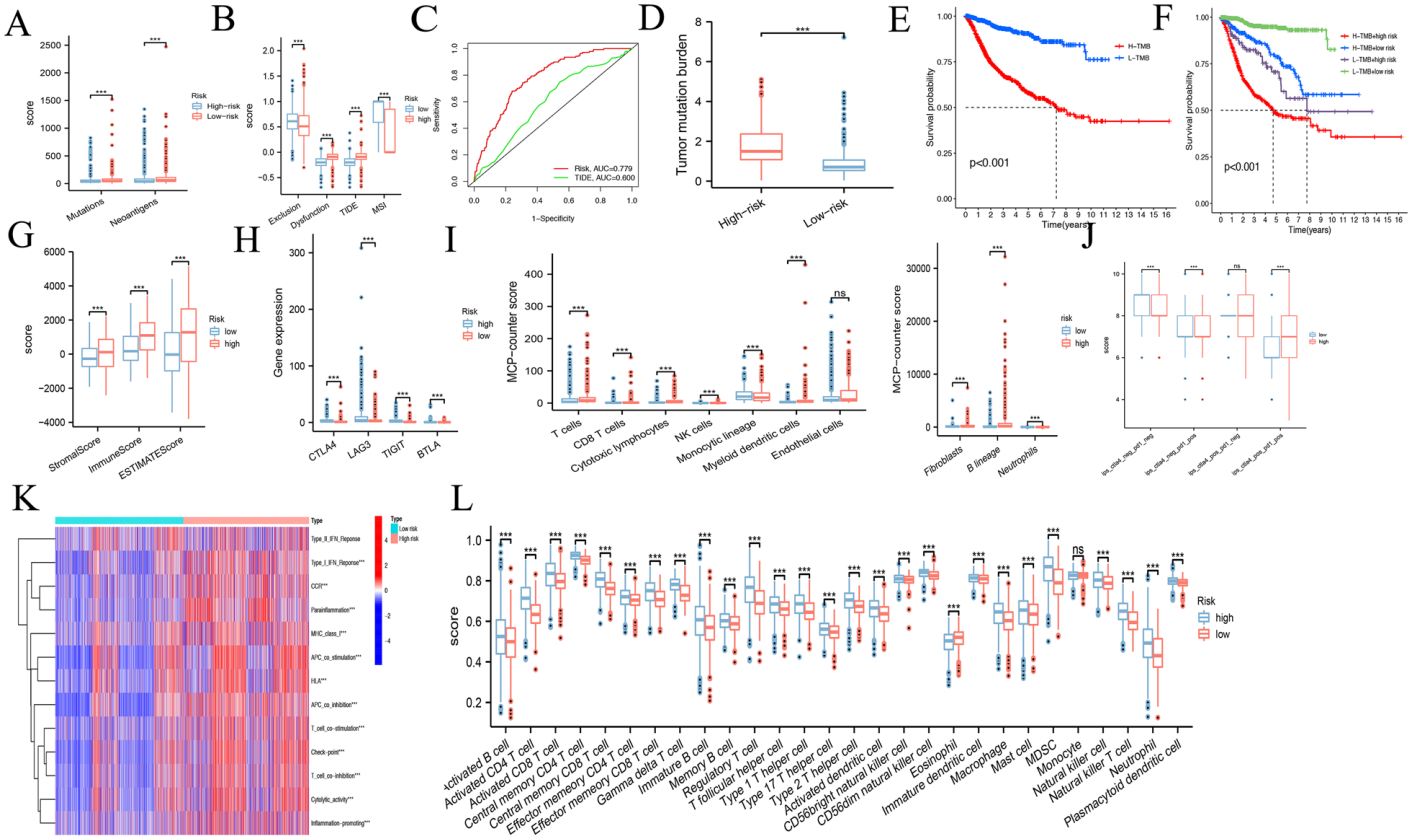
Immune landscape in low- and high-risk groups. **A** The number of mutations and neoantigens in low- and high-risk group patients. **B** The immune exclusion score, T cell dysfunction score, TIDE score, and MSI score in low- and high-risk groups. **C** ROC curves of TIDE score and risk. **D** TMB in low- and high-risk groups. **E** KM curves between high TMB and low TMB. **F** KM curves combined TMB and risk score. **G** Immune score, stromal score, and ESTIMATE score in low- and high-risk group. **H** Expression of immune checkpoint inhibitors in high- and low-risk groups. **I** Calculation of immune cell infiltration by MCP algorithm. **J** Difference of IPS with CTLA4- and PD-1-, CTLA4- and PD-1 + , CTLA4 + , and PD-1 and CTLA4 + and PD-1 + . **K** The result of GSVE between low- and high-risk. **L** ssGSEA results

We first compared the number of mutations and neoantigens between the high and low groups, and we found that the high-risk group met more mutations and neoantigens than the low-risk group (Fig. 7A). TIDE score is a new approach to evaluating the efficacy of immune checkpoint blockade (ICB). The TIDE algorithm is used to estimate the efficacy of tumors that respond to ICB treatment. High TIDE scores are associated with poor ICB treatment and short survival. To predict the potential for benefit from tumor immunotherapy, T cell dysfunction score, TIDE score, MSI score and immune exclusion score were calculated at the online website. The box plot showed that the T cell dysfunction score and TIDE score of the high-risk group were higher than those of the low-risk group, while the results of the MSI score and immune exclusion score were opposite (Fig. 7B). In terms of comparing the stability of the prognostic model and the prognostic value of the model in patients with immunotherapy, AUC was calculated for the prognostic model, TIDE score (Fig. 7C). The AUC of the risk score and TIDE score is 0.779 and 0.600, respectively, indicating that both had prognostic value. In turn, an AUC for the risk score greater than the TIDE score indicates that the risk model is more stable and more effective than the TIDE score. The “maftools” package was used to calculate the TMB of each patient, and the results showed that the TMB of the high-risk group was significantly higher than that of the low-risk group **(**Fig. 7D). Subsequently, we compared the overall survival rates between the high and low TMB groups, and the KM curve showed that the low-risk group had a better survival rate than the high-risk group (Fig. 7E). To further explore the relationship between TMB and OS, we combined risk scores with TMB to explore the correlation between TMB and the prognostic model. The results showed that the combined OS with low-risk scores and low TMB was significantly better than the other three groups (Fig. 7F). The “ESTIMATE” package was used to calculate the stromal score and the immune score and combined them to get the ESTIMATE score. The results showed that the tumor purity in the high-risk group was higher than that in the low-risk group (Fig. 7G). We also verified the expression of immune checkpoints in high- and low-risk groups, and the results showed that the expression of CTLA4, LAG3, TIGIT, and BTLA in the low-risk group was higher than that in the high-risk group (Fig. 7H). We performed the GSVA analysis to predict the immune cells, immune pathways and functions based on standardized gene expression data in the urinary system pan-cancer. The results showed that most immune-related processes were significantly different (Fig. 7I). Higher IPS was also exhibited by patients in the high-risk group compared with those in the low-risk (Fig. 7J). To explore the infiltration of immune cells, we used the MCP algorithm and ssGSEA analysis, and the results showed that the infiltration of high-risk immune cells was higher than that in the low-risk group (Fig. 7K, L). In summary, the high-risk group can be defined as a “hot” immune phenotype, associated with highly infiltrated antitumor immune cells and upregulated antitumor pathways.

### Drug sensitivity analysis based on the prognosis risk model

**Fig. 8:**
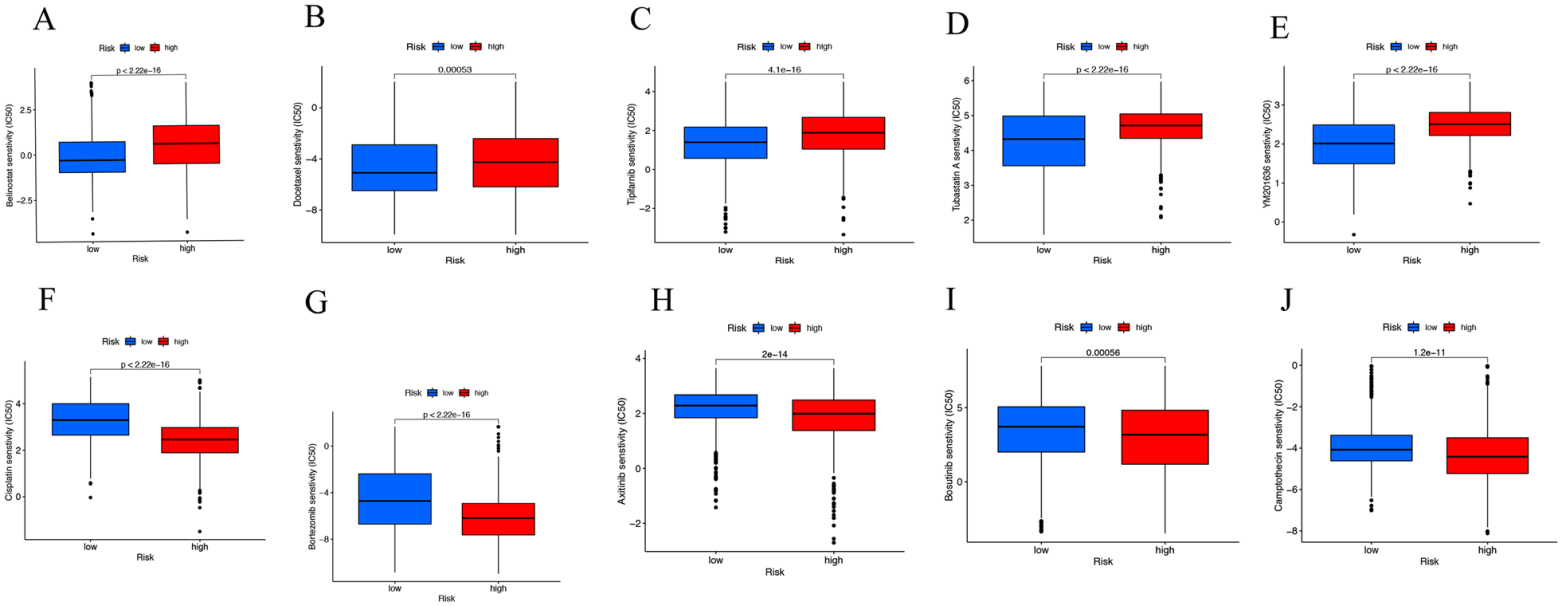
Drug sensitivity analysis in low- and high-risk groups. **A** Belinostat, **B** docetaxel, **C** tipifarnib, **D** tubastatin, **E** YM201636A, **F** Cisplatin, **G** bortezomib, **H** axitinib, **I** bosutinib, **J** camptothecin

We calculated the IC50 to compare the sensitivity of different targeted drugs in high- and low-risk groups. We found 146 drugs significant differences between the two groups, and here we showed five drugs which belinostat, docetaxel, tipifarnib, tubastatin, and YM201636A were more sensitive in the high-risk group (Fig. 8A–E), while cisplatin, 5-fluorouracil, axitinib, bosutinib, and camptothecin were more sensitive in the low-risk group (Fig. 8F–J).

### Functional enrichment analysis

**Fig. 9:**
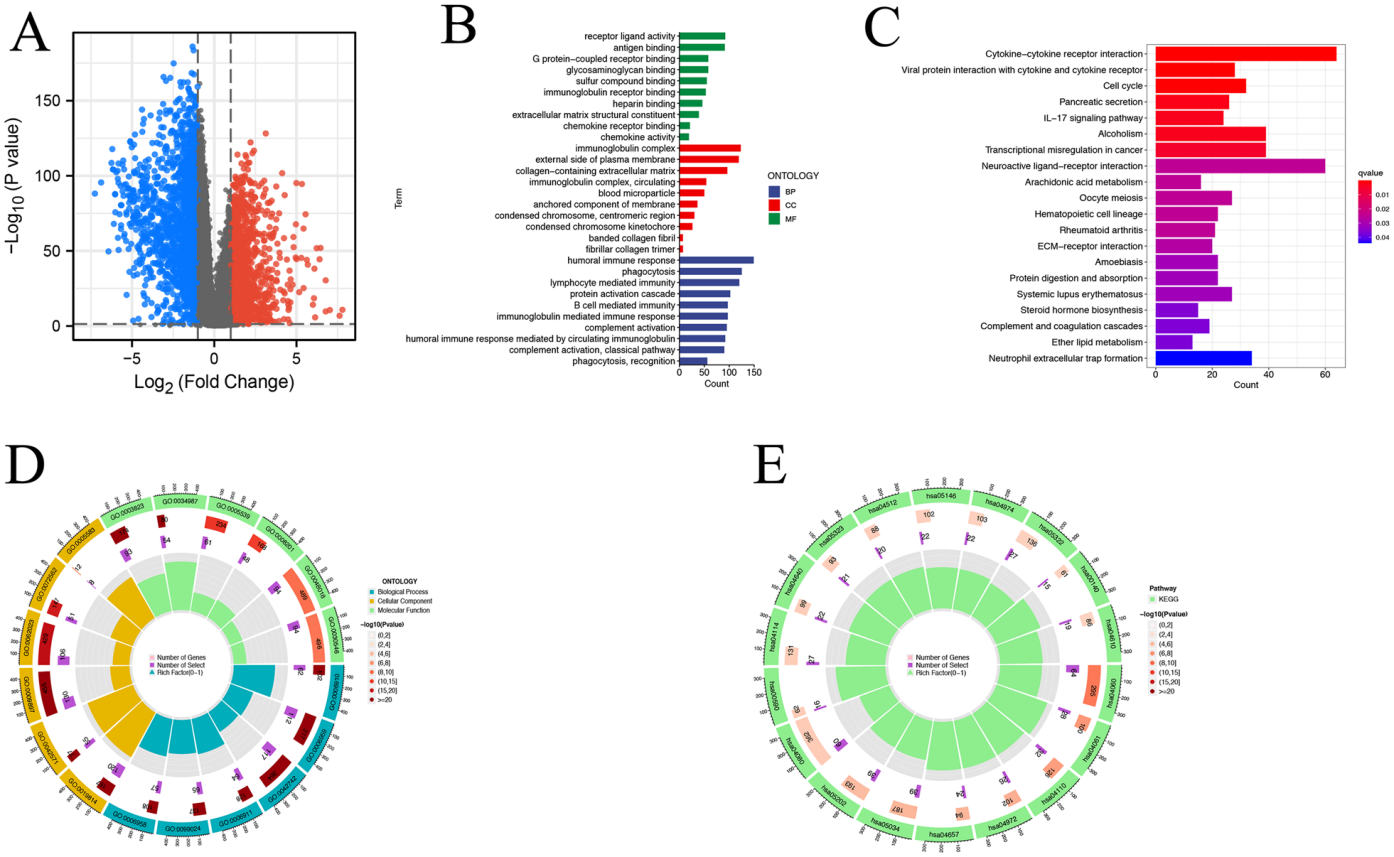
DEGs functional enrichment analysis. **A** The volcano plot showed the differentially expressed genes in the high and low groups. **B**, **C** GO enrich analysis. **D**, **E** KEGG enrich analysis

To determine the underlying function of the risk model, we identified the differentially expressed genes in both groups for Gene Ontology (GO) analysis and Kyoto Encyclopedia of Genes and Genomes (KEGG) analysis. We analyzed the differentially expressed genes (DEGs) in the high- and low-risk groups whthh logFC > 1, *P* value < 0.05 (Fig. 9A). DEGs were enriched in the specific binding of receptor-ligand biological processes, such as receptor-ligand activity, antigen binding, and G protein − coupled receptor binding. GO analysis also enriched immune-related cellular components and molecular functions, such as immunoglobulin complex, humoral immune response, and humoral immune response mediated by circulating immunoglobulin (Fig. 9B). The circle diagram incorporated the ID of GO and KEGG terms, genes, DEGs and the *P* value of DEGs which enriched on the corresponding to terms. (Fig. 9C, E). The two pathways with the most enriched differential genes are cytokine − cytokine receptor interaction and Neuroactive ligand−receptor interaction (Fig. 9D).

## Discussion

Bladder, prostate, and kidney cancer are the three most common tumors in the urinary system [1]. Although surgical treatment is the gold standard of urological tumor treatment, it is still prone to recurrence and metastasis after an operation, which seriously threatens the life and health of patients [19]. At present, medication is also the main treatment for urinary tumors [20]. The treatment of prostate cancer was mainly chemotherapy and new endocrine therapy [21]. Because renal carcinoma is not sensitive to chemotherapy, it is mainly treated with molecular targeting [22]. The development of prognostic markers for the early diagnosis of urinary system tumor is also particularly important as early symptoms are not easily detected in urological patients. Liquid biopsies are showing new promise in prostate cancer. The detection of prognostic marker in blood and other body fluids has become a new instrument for the early diagnosis, precise treatment, prognostic assessment and follow-up of patients with prostate tumor [23]. We also hope that liquid biopsies will be used in the treatment of many cancers as soon as possible.

Copper is controlled by the liver and is an essential cofactor in metabolism [24]. Existing studies have found that copper concentrations are stable in vivo, and once copper concentrations exceed a certain threshold, copper becomes toxic [25]. Then, the study had also shown that in addition to copper, Zinc dyshomeostasis was also associated with the development of cancer [26]. Cuproptosis is cell death based on excessive copper by targeting lipoylated TCA cyclin [5]. The current study has shown that have shown that although copper itself does not significantly affect mitochondrial respiration; this metal's toxicity is enhanced many times in cells that breathe actively. Therefore, cuproptosis may be applied to treat tumor with faster respiration rates and become a new type of biomarker and target for cancer treatment [9].

In our study, we analyzed the CRls in the three tumors and screened the co-expressed CRls in urinary pan-cancer. Finally, 12 CRls (BDNF−AS, WDFY3-AS2, FBXO30-DT, EDRF1-DT, AC106820.5, AC011477.2, SGMS1-AS1, CKMT2-AS1, AC015849.3 AL031670.1, AC096992.2, AL158212.3) and prognostic prediction models were obtained through three types of regression analyses. There are six CRLs have been reported as biomarkers in different cancer. lncRNA BDNF−AS was brain-derived neurotrophin factor antisense and downregulated in human prostate cancer. BDNF − AS may be a prognosis biomarker and inhibits the proliferation, invasion, migration, and EMT progression molecular intervening target in prostate cancer [27]. lncRNA WDFY3-AS2 acts as a ceRNA to enhance TIMP3 expression by acting as a sponge for Mir-21-5p, and Mir-221-3p [28]. AC106820.5 is also a CRLs and prognosis biomarker in head and neck squamous cell carcinoma [29]. lncRNA SGMS1-AS1 is under-expressed in lung cancer of lung adenocarcinoma cells by targeting Mir-106A-5p /MYLIP axis [30]. Enhancing the expression of CKMT2-AS1 may be an effective strategy to prevent the progression of colorectal cancer [31]. lncRNA AL031670.1 had been reported as a prognosis target in kidney clear cell carcinoma [32]. The remaining six lncRNAs have not been reported, but we discovered that they can be used as independent prognostic molecules in urinary system pan-cancer, and we speculate that they can be used as common new prognostic markers in urinary system pan-cancer.

To verify the precision of the risk model on the mechanism of the urinary tumor, we performed survival analysis, ROC curve, and mutation analysis. The highest area under the curve was observed in the risk score as well as the c-index [33]. This demonstrates the scientific validity and sensitivity of our model. To enable our risk model to be more valid in clinical diagnosis and treatment, we performed immunotherapy analysis as well as drug sensitivity analysis [34].

Tumor immunotherapy is a way to fight tumor by relying on the body's function, killing cancer cells and tumor tissues by activating the immune system, eliminating remaining lesions (local tumors or metastatic lesions), and preventing recurrence [35]. In recent years, immunotherapy has attracted much attention because of its small side effects and good efficacy [36]. Tumor immunotherapy is an ideal strategy for cancer treatment. Unlike conventional therapies, tumor immunotherapy is the activation of the body's innate immune system, which is a self-propagating cyclic process that leads to the accumulation of immune stimulators and enhanced T cell responses [37]. This cycle can be divided into seven main steps, starting from antigen release by cancer cells and ending with cancer cell killing. Current cancer immunotherapy typically focuses on two strategies: first, stimulating key players in the immune system, such as cancer vaccines, cytokine therapy, and adoptive T cell transfer; Second, the elimination or suppression of immunosuppressive factors, such as immune checkpoint blockade (ICB) therapy [19, 38, 39]. In recent years, the rapid development of cancer immunotherapy, especially represented by cancer vaccines and immune checkpoint blockade, has shown some exciting clinical responses [40].

It is well known that the accumulation of genetic mutations is the main cause of tumor formation [41]. Since genetic mutations provide a source of additional neoantigens and the neoantigens produce an antitumor immune response together with tumor-associated antigens [42]. This is consistent with our observation that both mutations and neoantigens were higher in the high-risk group than in the low-risk group. In order to better assess the benefit of immunotherapy in patients with urinary tumors, this study evaluated the difference in TIDE scores between high- and low-risk subgroups, suggesting that the TIDE scores were higher in the low-risk group. As the TIDE score is a response to sensitivity to immune checkpoint inhibitors, it had been shown that higher TIDE scores are associated with a greater likelihood of immune escape from tumors and are associated with poorer survival in patients treated with immune checkpoint inhibitors and less effective immunotherapy [43]. This study demonstrates that the high-risk group has a higher potential for immune escape, suggesting that the high-risk group is more likely to benefit from immunotherapy. The TIDE model is a computational method that simulates tumor immune evasion by integrating the expression characteristics of T cell dysfunction and T cell rejection, and can predict the clinical response to immunotherapy The TIDE model is a computational method that simulates tumor immune evasion by integrating the expression characteristics of T cell dysfunction and T cell rejection, and can predict the clinical response to immunotherapy Although TIDE can predict the response to immunotherapy, it does not predict the survival prognosis of patients and is focused on T cell functional status. Our model is more clinically relevant as it can predict both the response to immunotherapy and the survival prognosis of patients. In addition, we found not only significant differences in TMB across risk groups but also that patients in the high-risk group had lower survival than the low-risk group for both TMB and TMB combined with risk scores. This suggested that TMB may be associated with survival and prognosis [44].

Urinary system tumors are the most active immunotherapy species besides melanoma, as cytokine-based immunotherapy was the main treatment for advanced kidney cancer, BCG bladder infusion is the main treatment recommended for recurrence prevention after surgery for high-risk bladder cancer, and Sipuleucel-T, a prostate cancer vaccine, is the first therapeutic cancer vaccine approved by FDA so far [45–47]. Despite the obvious advantages of immunotherapy in urinary system tumors, different patients respond differently to immunotherapy due to the heterogeneity of tumors [48]. Thus, we analyzed stromal scores as well as immune cell infiltration in both risk groups. We used various methods to calculate multiple immune cell infiltrations, including ESTIMATE, MCP counter, and ssGSEA algorithm. Combined analysis showed that high-risk patients exhibited high immune scores and stromal scores. Also, various types of T cells, B cells, monocytes, and neutrophils had higher immune infiltration in the high-risk group. The above results suggested that patients in the high-risk group have higher tumor purity and are more suitable for immunotherapy.

Surgical resection remains the mainstay of treatment for urinary system tumors, yet about 30% of patients have distant metastases by the time of initial diagnosis [49, 50]. 20%-30% of patients who undergo surgery will have recurrence after surgery [51]. Since they are not sensitive to traditional radiotherapy, chemotherapy and hormone therapy, the clinical treatment strategies for urinary system tumors are very limited [52]. The main targeted drugs currently available for the treatment of urinary system tumors are sorafenib, sunitinib, and cisplatin [53–55]. We found that some of the targeted drugs showed different sensitivities to patients in the high- and low-risk groups. For example, docetaxel and tipifarnib may treat patients in the high-risk group. Meanwhile, cisplatin and axitinib may treat patients in the low-risk group. Cisplatin has been the foundation of bladder cancer treatment strategies for a long time, but about half of bladder cancer patients are truly suitable for cisplatin therapy, which is consistent with the results of drug sensitivity analysis showing that patients in the high-risk group are less sensitive to cisplatin. Studies have shown that FGFR 2 and FGFR 3 play an important role in bladder cancer. Unfortunately, sensitivity of cisplatin in patients with FGFR-altered bladder cancer were unclear. Hence, by calculating risk scores for bladder cancer patients with FGFR mutations, drug sensitivity to cisplatin can be further predicted [56]. This may provide a new thought for the treatment of bladder cancer. Consequently, an optimized approach based on CRLs prognostic model combining chemotherapy and targeted therapy may be relevant for the personalized treatment of urological patients.

Despite the relatively satisfactory results, our study still has some limitations. Since our data were obtained from the TCGA database, in vivo, and in vitro experiments are still needed to validate the risk score of the model and the biological function of CRLs.

In conclusion, our study constructed a prognostic risk model including 12 CRLs to accurately predict the prognosis of patients with urinary cancer and the efficacy of multiple immunotherapies. In the meantime, we have built the model into a more user-friendly website(https://l5035t-zhihui-ma.shinyapps.io/Urinarysystem/). This model may help patients with urinary cancer benefit from tumor immunotherapy.

## Data Availability

The data sets for our study are all from online databases, and the names of the databases are as follows: The Cancer Genome Altas database.
